# CRISPR/Cas9 Gene-Editing in Cancer Immunotherapy: Promoting the Present Revolution in Cancer Therapy and Exploring More

**DOI:** 10.3389/fcell.2021.674467

**Published:** 2021-05-20

**Authors:** Xuejin Ou, Qizhi Ma, Wei Yin, Xuelei Ma, Zhiyao He

**Affiliations:** ^1^Department of Biotherapy, State Key Laboratory of Biotherapy, West China Hospital, Sichuan University, Chengdu, China; ^2^Department of Thoracic Oncology, West China Hospital, Sichuan University, Chengdu, China; ^3^West China School of Medicine, Sichuan University, Chengdu, China; ^4^Department of Pharmacy, State Key Laboratory of Biotherapy and Cancer Center, National Clinical Research Center for Geriatrics, West China Hospital, Sichuan University, Chengdu, China

**Keywords:** CRISPR/Cas9, immunotherapy, TCR-T, tumor-infiltrating lymphocytes, CAR-T

## Abstract

In recent years, immunotherapy has showed fantastic promise in pioneering and accelerating the field of cancer therapy and embraces unprecedented breakthroughs in clinical practice. The clustered regularly interspaced short palindromic repeat (CRISPR)-associated protein 9 (CRISPR-Cas9) system, as a versatile gene-editing technology, lays a robust foundation to efficiently innovate cancer research and cancer therapy. Here, we summarize recent approaches based on CRISPR/Cas9 system for construction of chimeric antigen receptor T (CAR-T) cells and T cell receptor T (TCR-T) cells. Besides, we review the applications of CRISPR/Cas9 in inhibiting immune checkpoint signaling pathways and highlight the feasibility of CRISPR/Cas9 based engineering strategies to screen novel cancer immunotherapy targets. Conclusively, we discuss the perspectives, potential challenges and possible solutions in this vivid growing field.

## Introduction

In recent years, cancer immunotherapy, including immune checkpoint blockades and adoptive T cell therapy (ACT), has experienced incredible success in various types of cancer. Immunotherapies mainly function by relieving tumor-induced immunosuppression and re-boosting anti-cancer immunity (O’Donnell et al., 2019). Immune checkpoint blockades, such as anti-PD1/PD-L1 antibody and anti-CTLA-4 antibody, inhibit immunosuppressive signals and promote T cells reinvigoration. Adoptive T cell therapy, including tumor infiltrating lymphocytes (TILs) therapy, transgenic T cell receptor (TCR)-T cell therapy and chimeric antigen receptors (CAR)-T cell therapy, functions by increasing the number of tumor-reactive T cells and directly against tumor cells. Although significant and durable clinical responses have been resulted from cancer immunotherapy in certain cancer types, unfortunately, most patients fail to benefit from immunotherapy due to intrinsic and adaptive tumor resistance. Therefore, additional and novel immunotherapies are in urgent need.

CRISPR/Cas9, as a versatile gene-editing technology, has been extensively applied in cancer research. Since its first application as a genome-editing tool in mammalian cells in 2013, the use of CRISPR/Cas9 system has been rapidly expanded owing to its high flexibility and efficiency (Cong et al., 2013). CRISPR/Cas9 has been widely used in establishing cancer models (Platt et al., 2014; Tuveson and Clevers, 2019), verifying essential genes as druggable targets (Evers et al., 2016; Tzelepis et al., 2016), investigating the mechanism of drug resistance (Pettitt et al., 2018; Wei L. et al., 2019), comprehensively understanding the function of gene non-coding regions (Zhu et al., 2016; Esposito et al., 2019), and so on. An in-depth discussion of CRISPR/Ca9 in cancer research is has been recently reviewed in detail elsewhere (Zhan et al., 2019), which will not be covered in this review. The combination of CRISPR/Cas9 and cancer immunotherapy, the two revolutionary technologies in cancer research and treatment, may further broaden the application of immunotherapy to more cancer patients. In this review, we summarized recent developments of CRISPR/Cas9 technology in cancer immunotherapy, involving the construction of CAR-T cells, designing of TCR-T cells, inhibiting immune checkpoint signaling pathways, and screening for new druggable targets in immunotherapy.

## Application of CRISPR/Cas9 System in CAR-T Cell Immunotherapy

**FIGURE 1:**
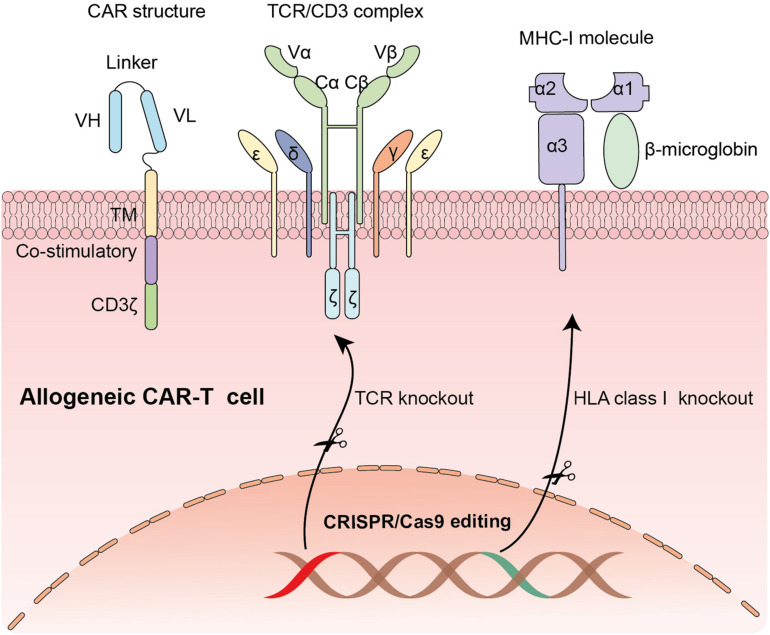
The application of CRISPR/Cas9 system in editing CAR-T cells: Knocking out endogenous TCR locus and MHC-1 molecule to generate universal CAR-T cells.

Recently, genetically modified T cells that express chimeric antigen receptors (CAR T-cells) have shown unprecedented efficacy in hematological malignancies. A CAR structure is comprised of three parts: an extracellular antigen recognizing domain usually a single-chain variable fragment (scFv) derived from an antibody, a transmembrane domain, and an intracellular signaling transduction domain containing CD3ζ chain with or without costimulatory molecules (Figure 1). One of the most successful clinical trials is applying anti-CD19 CAR-T cells in B-cell malignancy (Maude et al., 2014). Because of the striking clinical efficacy of CAR-T cells, Kymriah and Yescarta, two types of anti-CD19 CAR-T cells, have been approved by the U.S. Food and Drug Administration (FDA) to treat pediatric/young adult B lymphoblastic leukemia (B-ALL) and adult diffuse large B cell lymphoma (DLBCL). In July 2020, Tecartus was approved for the treatment of adult patients with mantle cell lymphoma (MCL). In February 2021, Breyanzi, the fourth CAR-T therapy for adults with relapsed and refractory large B cell lymphoma, was approved by US FDA. Till now, a large number of registered clinical trials are springing up. Novel constructions and various applications of CAR-T cells are developed at a rapid pace (Jackson et al., 2016; Zhang C. et al., 2017; Labanieh et al., 2018).

Despite such impressive clinical results have been achieved, plenty of patients are unable to benefit from T-cell therapy due to several reasons. First, the personalized approach of manufacturing T-cells is time-consuming and costly, which impedes many patients, especially with rapidly progressive diseases, to make the most of this immunotherapy. Second, during the production process, it is hard to generate enough high-quality T-cells from lymphopenic patients in poor condition. Even if patients get enough immune cells, these cells may fail to complete the whole manufacturing process. Moreover, the risk of manufacturing T cells always exists (Torikai and Cooper, 2016). A patient with B cell leukemia was reported relapsing 9 months after receiving anti-CD19 CAR T-cell infusion due to unintentionally transduction of CAR gene into a single leukemic B cell (Ruella et al., 2018). Finally, heterogeneity among autologous CAR-T products contributes to unpredictable and variable clinical activity.

## Generation of Universal Allogeneic CAR-T Cells by CRISPR/Cas9

To overcome the barriers of limiting wide application of CAR-T cell therapy, multiple strategies have been developed. One of the most feasible and durable approaches is to generate allogeneic universal CAR-T cells from healthy donors (Depil et al., 2020). Compared with autologous CAR-T cells, “off-the shelf” allogeneic CAR-T cells have many potential advantages, including immediate availability of cryopreserved CAR-T cells for patients in urgent need, enough quantity for first infusion or re-dosing and possible standardization of CAR-T cells producing (Depil et al., 2020). Whereas considering the presence of endogenous HLA and TCR on donor’s T lymphocytes, the biggest challenge of universal products is the potential risk of alloreactivity (host versus graft response) and graft-versus-host disease (GVHD) (Figure 1). With the advances of Gene editing technology, eradication of endogenous TCR can be achieved. Hiroki Torikai and colleagues generated CD19 specific CAR-T cells with disruption of endogenous TCR via zinc finger nucleases (ZFNs) to reduce graft-versus-host responses. The genetically modified CAR-T cells showed an expected specificity of CD19 antigen with no responding to TCR stimulation (Torikai et al., 2012). Similarly, transcription activator-like effector nucleases (TALENs), another widely used gene-editing tool, have also been used in producing universal CAR-T cells by knocking out αβ chains of TCR (Poirot et al., 2015). However, generation of fully allogeneic CAR-T cells requires simultaneous knockout of TCR and HLA molecules and transduction of CAR. An efficient and precise gene-editing technique with the unique capability to achieve multiplexed genome engineering is needed.

Compared with ZFNs and TALENs, CRISPR/Cas9 has more applications in producing allogeneic CAR-T cell due to its excellent flexibility and high effectiveness. CRISPR/Cae9 system can simultaneously and efficiently knock out multiple gene loci. TCR^–^ HLA class I^–^,Fas^–^TCR^–^ HLA class I^–^,PD1^–^ TCR^–^ HLA class I^–^ allogeneic universal T cells can be easily yielded via a one-shot CRISPR protocol by incorporation of multiple guide RNAs in a CAR lentiviral vector (Ren et al., 2017b; Choi et al., 2019). Ren et al. used CRISPR/Cas9 to generate CAR-T cells simultaneously deficient in endogenous TCR, HLA-I and PD-1, which shows potent antitumor activity *in vitro* and in animal models (Ren et al., 2017a). Besides, a uniform CAR expression was generated by inserting a CD19 specific CAR into the T-cell receptor α constant (TRAC) locus via CRISPR/Cas9 genome editing (Eyquem et al., 2017). The edited cells vastly outperformed the conventional CAR-T cells with enhanced anti-tumor activity *in vitro* and in mouse models with acute lymphoblastic leukemia (Eyquem et al., 2017). While the safety and efficacy of CRISPR/Cas9-edited universal CAR T cells *in vivo* needs to be further tested in clinical studies. Currently, eight relevant clinical trials are going on (Table 1).

**TABLE 1:** Registered clinical trials using CRISPR/Cas9 modified universal CAR-T cells for treatment of malignancies.

Row	Identifier	Phase	Status	Condition or diseases	Interventions	Genes knockout	Estimated enrollment
1	NCT03545815	I	Recruiting	Solid tumor, adult	Universal anti-mesothelin CAR-T cells	PD-1, TCR	10 patients
2	NCT03398967	I/II	Recruiting	B cell leukemia/B Cell Lymphoma	Universal dual specificity CD19 and CD20 or CD22 CAR-T cells	Unknown	80 patients
3	NCT03166878	I/II	Recruiting	B cell leukemia/B cell lymphoma	Universal anti-CD19 CAR-T cells	TCR, B2M	80 patients
4	NCT04502446	I	Recruiting	T or B cell malignancies	Universal anti-CD70 CAR-T cells (CTX130)	Unknown	45 patients
5	NCT04244656	I	Recruiting	Multiple myeloma	Universal anti-BCMA CAR-T cells (CTX120)	Unknown	80 patients
6	NCT04438083	I	Recruiting	Renal cell carcinoma with clear cell differentiation	Universal anti-CD70 CAR-T cells (CTX130)	Unknown	105 patients
7	NCT04035434	I	Recruiting	B-cell Malignancy Non-Hodgkin lymphoma	Universal anti-CD19 CAR-T cells (CTX110)	Unknown	131 patients
8	NCT04637763	I	Recruiting	B Cell Non-Hodgkin lymphoma	Universal anti-CD19 CAR-T cells (CB-010)	Unknown	50 patients

*From clinicaltrials.gov, accessed 02-26-2021. PD-1: programmed death-1; TCR: T cell receptor; B2M: beta 2 microglobulin.*

## Enhancing CAR-T Cell Function via CRISPR/Cas9

**FIGURE 2:**
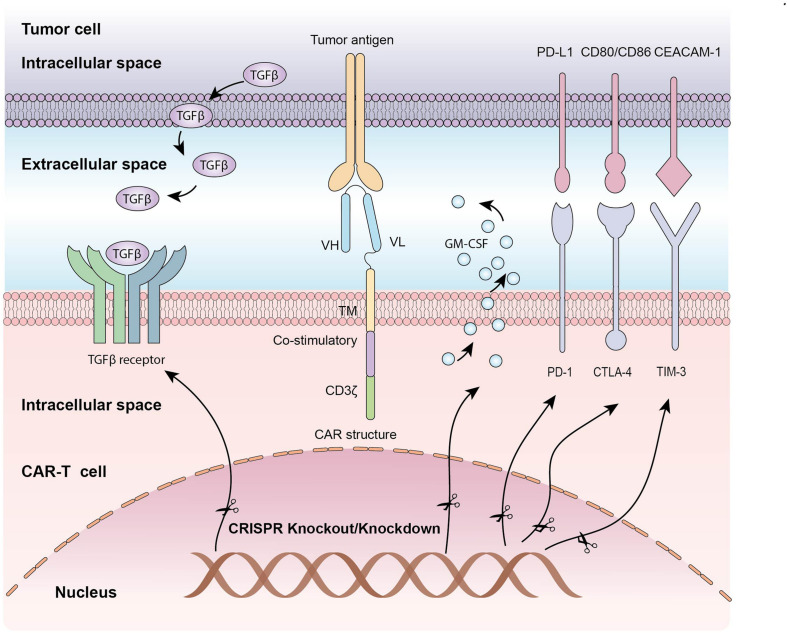
The application of CRISPR/Cas9 system in editing CAR-T cells: Knocking out inhibitory molecules to enhance function of CAR-T cells.

Despite remarkable success have been achieved in the treatment of hematological malignancies, CAR-T adoptive cell therapy have been floundered in many patients, to some extent due to immunosuppressive tumor microenvironment and T cell exhaustion (Cherkassky et al., 2016). Because of the established role of co-inhibitory molecules, such as PD-1, CTLA-4, LAG-3 and TIM-3 in T cells dysfunction, CRISPR/Cas9 system has also been applied to disrupt these inhibitory genes to enhance CAR-T cell function (Table 2 and Figure 2). CRISPR/Cas9-mediated PD-1 depletion was proven to augment the ability of CAR-T cells in killing tumor cells *in vitro* and clearing PD-L1^+^ tumor xenografts *in vivo* (Rupp et al., 2017; Choi et al., 2019). In addition to co-inhibitory genes, Diacylglycerol Kinase (DGK) ablation in CAR-T cells resulting in improvement of anti-tumor immunity (Jung et al., 2018). Knocking out granulocyte-macrophage colony-stimulating factor (GM-CSF) gene was demonstrated to enhance CAR-T cells function as well as reduce the risk of cytokine release syndrome (CRS) and inflammation (Sterner et al., 2019). Studies have also confirmed that knocking down the endogenous TGF-β receptor II (TGFBR2) in CAR T cells with CRISPR/Cas9 technology could decrease the exhaustion of CAR-T cells and increase solid tumor-killing efficacy both *in vitro* and *in vivo* (Tang et al., 2020). Moreover, eradicating CD7 and TRAC in CAR T cells by CRISPR/Cas9 increased the efficacy to treat T cell acute lymphoblastic leukemia (T-ALL) (Cooper et al., 2018).

**TABLE 2:** Representative targets of CAR-T cells engineered by CRISPR/Cas9 system.

Target genes	Target cells	Cancer cell lines	Tools	CRISPR/Cas9 delivery ways	Results	Year/Journal	References
LAG-3	Anti-CD19 CAR-T cells	Raji, K19, K562	CRISPR/Cas9	Electroporation	LAG-3 knockout CAR-T cells display comparable effector functions to standard CAR-T cells	2017/Front.Med.	Zhang Y. et al., 2017
PD-1	Anti-CD19 CAR-T cells	K562	CRISPR/Cas9 RNP	Electroporation	PD-1 disruption augmented anti-tumor ability of CAR-T cells	2017/Sci Rep	Rupp et al., 2017
DGK	anti-EGFRvIII CAR-T cells	U87 MG glioblastoma cell line	CRISPR/Cas9 RNP	Electroporation	DGK knockout rendered CAR-T cells resistant to soluble immunosuppressive factors	2018/Cancer Res	Jung et al., 2018
GM-CSF	Anti-CD19 CAR-T cells	NALM6	CRISPR/Cas9	Lentiviral vector	GM-CSF knockout CAR-T cells exhibited decreased expression of GM-CSF with normal function and enhanced anti-tumor activity	2019/Blood	Sterner et al., 2019
TGFBR2	Anti-mesothelin CAR T cells	CRL5826	CRISPR/Cas9	Electroporation	TGFBR2 edited CAR-T cells had better *in vivo* elimination of tumor cells, with an increased proportion of memory T cell subsets	2020/JCI Insight	Tang et al., 2020

*LAG-3: lymphocyte activation gene 3 protein; PD-1: programmed death-1; DGK: diacylglycerol kinase; GM-CSF: granulocyte-macrophage colony-stimulating factor; TGFBR2: TGF-β receptor II.*

## Application of CRISPR/Cas9 System in T Cell Receptor (Tcr)-Based Adoptive T Cell Therapy

**FIGURE 3:**
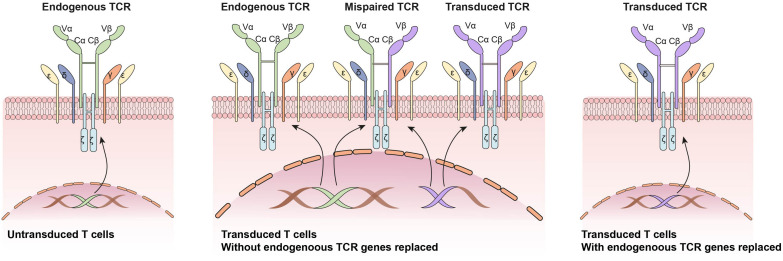
Application of CRISPR/Cas9 system in engineered T cell receptor (TCR)-based adoptive T cell therapy: Knocking out endogenous TCRs to avoid mixed TCR dimer formation and improve transduced TCR expression.

It is known that CAR-T cells play limited roles in solid tumors. The main causes include lack of tumor specific antigens, heterogeneity of tumor antigens and tumor microenvironment suppression. Compared with CAR-T immunotherapy, engineered TCR-T cell therapy holds greater promise for targeting a wider range of antigens and thereby enlarge the scope to treat cancers (Morris and Stauss, 2016). The T cell receptor is an antigen recognition structure expressed on the membrane of T cells. The TCR is a heterodimer consisting of TCRα chain and TCR β chain. Both chains contain a variable antigen binding region, extracellular constant region and transmembrane region. The constant regions of TCRα/β chain and CD3 chains (γ, δ, ζ, ε) form the TCR/CD3 complex (Figure 3). The TCR/CD3 complex recognizes tumor antigens in a major histocompatibility complex (MHC)-dependent manner. Natural TCR specificities of tumor infiltrating lymphocytes (TILs) have been successfully exploited as adoptive cell therapy, and remarkable clinical responses have been achieved in several solid tumors, such as melanoma (Rosenberg et al., 1988), cholangiocarcinoma (Tran et al., 2014), breast cancer (Zacharakis et al., 2018) and papillomavirus-associated cervical cancer (Doran et al., 2019). While adoptive transfer of T cells with engineered tumor-specific TCRs has also shown promising therapeutic potential in several cancers, including melanoma (Robbins et al., 2011; Chodon et al., 2014), sarcoma (Robbins et al., 2011), and multiple myeloma (Mastaglio et al., 2017).

One of the major issues related to the generation of TCR-T is the pre-existing endogenous TCRs on the recipient T cells. The endogenous TCRs competes with transgenic TCRs for CD3 association and surface expression. Besides, there is also potential for mixed TCR dimer formation by mispairing between endogenous and transgenic TCRs. To circumvent these problems, CRISPR/Cas9 system was applied to replace endogenous TCRs α and β genes with artificial tumor-specific TCR sequence (Mastaglio et al., 2017; Legut et al., 2018; Morton et al., 2020; Figure 3). Knocking out endogenous TCRs leads to improved expression and function of transgenic TCRs compared with conventional TRC-T cells (Legut et al., 2018). Besides, anti-tumor responses of CRISPR/Cas9 modified T cells was enhanced in animal models (Roth et al., 2018). To reduce the risk of mispairing, another strategy is to transduce a stabilized Vα/Vβ single-chain TCRs (Sc-TCRs) (Aggen et al., 2012). The combination of Sc-TCRs transduction and CRISPR disruption was reported to almost completely eliminate TCR mispairing (Xue et al., 2020). The first clinical trial testing the safety and feasibility of CRISPR/Cas9 editing TCR-T cells in patients with refractory cancer was reported in 2020 (Stadtmauer et al., 2020). Patients’ autologous T cells were isolated and engineered by lentiviral transduction to express an TCR-specific for NY-ESO-1 and LAGE-1. Then the endogenous TCRs and PD-1 genes were disrupted by CRISPR/Cas9 system. The CRISPR modified T cells were expanded *in vitro* and re-fused to three patients. Two patients experienced a stable disease, and the other patient experienced disease progression (Stadtmauer et al., 2020).

In addition to knock out endogenous TCR genes, CRISPR/Cas9 system has also been used to identify a TCR that recognized and killed cancer cells in MHC-independent manner (via monomorphic MHC class I related protein, MR1) (Crowther et al., 2020).

## Inhibition of Immune Checkpoint Signaling Pathway

In the past decade, immunotherapy has revolutionized cancer treatment by activating immune system to eliminate malignant tumor cells (Mellman et al., 2011; Page et al., 2014; Zarour, 2016). Although tumor cells can be immunogenic, and the presence of CD8^+^ tumor infiltrating lymphocytes was regarded as a positive prognostic factor in multiple solid tumors (Azimi et al., 2012; Bremnes et al., 2016; Pollari et al., 2018), the immune system often fails to eradicate tumor cells *in vivo*. The paradoxical coexistence of tumor-reactive T cells and tumor cells may arise from T cell exhaustion due to persistent antigen exposure and immunosuppressive factors in the tumor microenvironment (Schietinger and Greenberg, 2014; Pauken and Wherry, 2015; Wherry and Kurachi, 2015; Zarour, 2016). Gene profiling and phenotypical studies in human and mice have shown that exhausted tumor-infiltrating T lymphocytes typically express high level of inhibitory receptors including cytotoxic T lymphocyte antigen-4 (CTLA-4, CD152), programmed death-1 (PD-1, CD279), lymphocyte activation gene 3 protein (LAG3), T cell immunoglobulin domain and mucin domain-containing protein 3 (TIM-3, HAVCR2), 2B4 (CD244), CD160, TIGIT, and many other inhibitory molecules (Fourcade et al., 2012; Qin et al., 2019). In recent years, these immune checkpoint molecules have arisen a wide attention in cancer research due to their critical roles in anti-tumor immunity.

Once antigens are recognized by TCR/CD3 complex, CD28, as a co-stimulatory molecule, can strongly amplify TCR signals to activate T cells upon binding to CD80 (B7-1) or CD86 (B7-2) (Krummel and Allison, 1995; Rowshanravan et al., 2018). Whereas, CTLA-4, as a homologous receptor of CD28, is expressed exclusively on both CD4 + and CD8 + T cells and mediates an opposing function in T cell activation by delivering inhibitory signals upon binding to the same ligands as CD28 (Krummel and Allison, 1995). Targeting CTLA-4 by monoclonal antibody or genetic tool increases the availability of ligands to CD28, allowing to enhance T cell activation. Accordingly, Ipilimumab, a recombinant human IgG1 monoclonal antibody to blockade CTLA-4, was developed to boost patents’ immunity to eliminate tumor cells (Leach et al., 1996; Hodi et al., 2010). Ipilimumab, used as a single agent or in combination with gp100 peptide vaccine, as compared with gp100 vaccine alone, improved overall survival of patients with metastatic melanoma (Hodi et al., 2010). It was approved by US FDA for the treatment of metastatic melanoma in March 2011. Anti-CTLA-4 inhibitors have also been extensively investigated in many other cancers, such as non-small cell lung cancer, breast cancer, prostate cancer, and so on (Boutros et al., 2016; Page et al., 2016; Beer et al., 2017). In addition to using antibody to blockade CTLA-4, CRISPR/Cas9 has been applied to knockout CTLA-4. Shi et al. (2017) and Zhang et al. (2019) knocked out CTLA-4 in cytotoxic T lymphocytes (CTLs) using CRISPR/Cas9 system, and found that CTLA-4 disruption, as compared with control groups, could increase TNF-α and IFN-γ secretion and enhance anti-tumor activity of CTLs.

PD-1/PD-L1 immune checkpoint axis is another T cell function regulating pathway, upon TCR binding to MHC-peptides presented by antigen presenting cells (Keir et al., 2008). It has been found that PD-1 is expressed on a variety of activated immune cells, including T cells, monocytes, dendritic cells and so on (Alsaab et al., 2017). While its ligands PD-L1 and PD-L2 are expressed on tumor cells and antigen presenting cells. The interaction between PD-1 and PD-L1 suppresses T cell activation and function, and results in T cell exhaustion (Alsaab et al., 2017; Goodman et al., 2017). Inhibiting PD-1/PD-L1signal pathway using monoclonal antibody can reinvigorate T cell function. And anti-PD-1 antibody, nivolumab and pembrolizumab, have shown impressive anti-tumor responses in diverse malignancies, such as melanoma, non-small cell lung cancer, head and neck squamous cell carcinoma, and metastatic urothelial carcinoma (Brahmer et al., 2015; Robert et al., 2015; Ferris et al., 2016; Sharma et al., 2017). Therefore, the disruption of inhibitory genes may be a potential alternative to blockade immunotherapy. CRISPR/Cas9 has recently been applied to disrupt PD-1 expression on human peripheral blood T cells, CAR-T cells, and antigen specific cytotoxic T lymphocytes (CTLs) (Su et al., 2016, 2017; Ren et al., 2017a; Rupp et al., 2017; Shao et al., 2017; Guo et al., 2018; Zhang et al., 2018; Nakazawa et al., 2020). Su et al. (2016, 2017) reported the knockout of PD-1 by electroporation of plasmids encoding CRISPR/Cas9 system was technically feasible and did not affect the viability of T cell *in vitro*. Besides, IFN-γ secretion and anti-tumor cytotoxicity of gene modified T cells was enhanced. A recently completed clinical trial have reported that PD-1 disrupted T cells by CRISPR/Cas9 were safe and feasible, but lack of efficacy in patients with non-small cell lung cancer (Lu et al., 2020). Another trail evaluating the safety of PD-1 knockout T cell based on CRISPR/Cas9 system in patients with advanced esophageal cancer (NCT03081715) has also been registered and completed. While three other registered trials applying the same concept of PD-1 disrupted T cells in the treatment of muscle-invasive bladder cancer (NCT02863913), castration resistant prostate cancer (NCT02867345) and renal cell carcinoma (NCT02867332) have been withdrawn.

In addition to PD-1 knockout, disruption of PD-L1 in tumor cells has also been demonstrated to improve the efficacy of cancer immunotherapy. Tu et al. designed a novel type of weak acidity-responsive nanoparticles featured with CRISPR/Cas9-Cdk5 plasmid (Cas9-Cdk5) and paclitaxel (PTX). PTX encapsulated in the nanoparticles could induce immunogenic cells death and reduce suppressive immune cells. While the CRISPR/Cas9 plasmids could specifically target cyclin-dependent kinase 5 gene to mediate PD-L1 attenuation on tumor cells, so as to enhance anti-tumor immune response (Tu et al., 2020). Besides, another study conducted by Zhao et al. (2020) constructed a photoswitched CRISPR/Cas9 system to target PD-L1 gene. Under light irradiation, this system could efficiently knock out PD-L1 gene not only in bulk cancer cells but cancer stem-like cells as well.

## Novel Targets Screening for Cancer Immunotherapy

Tumor mutations can give rise to neoantigens and elicit anti-tumor immunity. However, genetic alterations can also induce immunotherapy resistance (Patel et al., 2017). For instance, tumor cells deficient in interferon-receptor signaling (JAK1/2) and antigen-presenting pathway (beta-2-microglobulin, B2M) have been reported to be associated with resistance to PD-1 blockade immunotherapy (Zaretsky et al., 2016). CRISPR/Cas9 system as a formidable large-scale gene screening toolkit, is developed to identify critical genes and new targets in cancer treatment (Liu et al., 2020). Various strategies of CRISPR screen for novel immunotherapy (IO) targets discovery have been used, including targeting antigen processing and presentation, IFN-γ pathway, TNF signaling, epigenetic regulators, and PD-L1 regulators in tumor cells, and metabolic regulators in T cells (Table 3).

**TABLE 3:** Summary of new targets identified by CRISPR/Cas9 screening.

Target pathways	Target cells	Immune selective pressure	Target library	CRISPR/Cas9 delivery ways	Significant targets	Year/Journal	References
Antigen processing and presentation pathway IFN-γ pathway	Melanoma cell lines	PD-1 blockade	9,872 sgRNAs targeting 2,368 genes	Lentiviral vector	PTPN2	2017/Nature	Manguso et al., 2017
Antigen processing and presentation pathway IFN-γ pathway	Melanoma cell lines	NY-ESO-1 specific TCR-T cells	123,411 sgRNAs targeting 19,050 genes	Lentiviral vector	APLNR	2017/Nature	Patel et al., 2017
Chromatin regulators	B16F10 melanoma cells	Pmel-1 T cells OT-I T cells	>100 genes	Lentiviral vector	PBAF, PBRM1, ARID2	2018/Science	Pan et al., 2018
T cell activation regulators	Jurkat T cells	/	250,000 total sgRNAs targeting every unique Refseq annotated (hg19) protein-coding genes	Lentiviral vector	FAM49B	2018/Proc Natl Acad Sci U S A	Shang et al., 2018
T cell stimulation regulators	Primary human CD8^+^ T cells	/	77,441 sgRNAs targeting 19,114 genes	single guide RNA (sgRNA) lentiviral infection with Cas9 protein electroporation	SOCS1, TCEB2, RASA2, CBLB	2018/Cell	Shifrut et al., 2018
Regulators of tumor infiltration and degranulation	Mouse and human CD8 T cells	/	128,209 gene-specific sgRNAs	Lentiviral vector	DHX37	2019/Cell	Dong et al., 2019
Antigen processing and presentation pathway IFN-γ pathway	B16-F10 melanoma cells	Mouse NK cells	Brie genome-wide sgRNA library	Lentiviral vector	Jak1	2019/Cell Rep	Freeman et al., 2019
IFNg-independent signaling pathway	IFNGR1-deficient melanoma cells	MART-1 T cells	GeCKO library	Lentiviral vector	TRAF2	2019/Cell	Vredevoogd et al., 2019
T cell metabolic regulators	OT-1 T cells	/	3,017 metabolism related genes	Lentiviral vector	Regnase-1	2019/Nature	Wei J. et al., 2019
Cell membrane targets	Mouse CD8 T cells	/	1,658 mouse membrane protein encoding genes	AAV vector and Sleeping Beauty transposon system	Mgat5, Emp1 Lag3, PDIA3	2019/Nat Biotechnol	Ye et al., 2019
Gene regulatory programs in Foxp3 expression	Primary mouse Tregs	/	Brie library	Retroviral vector	Usp22, Rnf20	2019/Nature	Cortez et al., 2020
Epigenetic regulators	Kras^G12D^/Trp^53–/–^ lung cancer cells	Anti–PD-1 antibody	524 epigenetic regulators genes	Lentiviral vector	Asf1a	2020/Cancer Discov	Li et al., 2020
PD-L1 regulators	Human lung adenocarcinoma cell line H358 cells	/	The human GeCKO version 2 library	Lentiviral vector	eIF5B	2020/Nat Cancer	Suresh et al., 2020

To target antigen processing and presentation and IFN-γ pathway, Manguso et al. created a library consisting of 9,872 sgRNAs targeting 2368 genes, then delivered the library into B16 melanoma cells via lentiviral infection. After transduction, tumor cells were purified and implanted into mice that were then treated with immunotherapy, so as to apply immune selective pressure on the transduced tumor cells. In parallel, the library-transduced tumor cells were transplanted into Tcra-/- mice in order to generate animal models without immune selective pressure. After about 12–14 days, tumor tissues were collected. The library representation in tumors from wild type mice or Tcra-/- mice were compared. In this study, they found that deletion of protein tyrosine phosphatase PTPN2 sensitized tumor cells to immunotherapy by enhancing IFN-γ signaling pathway (Manguso et al., 2017). Patel et al. (2017) transduced NY-ESO-1^+^ Mel624 melanoma cells with a genome-scale CRISPR library of 123,411 sgRNAs, and constructed NY-ESO-1 antigen specific TCR-T cells. Then the transduced melanoma cells were co-incubated with NY-ESO-1 antigen specific TCR-T cells. They found antigen presentation and IFN-γ pathway related genes, such as HLA-A, B2M, TAP1, TAP2, and TAPBP were among the most critical genes in the screen. Besides, the functional loss of APLNR reduced the efficacy of immunotherapy by interacting with JAK1 and modulating IFN-γ responses in tumors (Patel et al., 2017).

By targeting TNF signaling, metabolic protein 2-aminoethanethiol dioxygenase (Ado) was found to modulate sensitivity of tumor cells to TNF released by cytotoxic T cells (Kearney et al., 2018). Vredevoogd et al. reported that selectively inactivation of TNF pathway, such as the ablation of TRAF2,sensitized tumor cells to T cell attack (Vredevoogd et al., 2019). To identify critical epigenetic regulators in anti-tumor immunity, a sgRNA library focused on epigenetic regulators were constructed and CRISPR screening was performed in a Kras^G12D^/Trp53^–/–^ lung adenocarcinoma model. Loss of the histone chaperone Asf1a was found to sensitize tumors to anti-PD-1treatment (Li et al., 2020). In order to identify important regulators of PD-L1 expression. Suresh et al. (2020) performed a genome wide loss-of-function CRISPR screen in human lung cancer cells. They demonstrated that heme biosynthesis pathway was a key regulator of PD-L1 expression, and impairment of heme production would activate integrated stress response (ISR), resulting in enhanced PD-L1 expression. While the translation initiation factor eIF5 was required in ISR-dependent PD-L1 expression. Targeting eIF5 might be potentially therapeutic. In addition to targeting genes in tumor cells, Jun Wei and colleagues constructed two sub-libraries of sgRNAs targeting 3,017 metabolism related genes in T cells to investigate the roles of metabolism-associated factors in T cell immunity. They demonstrated that CD8 T cells could be reprogramed to long-lived effector cells by knocking out Regnase-1. Regnase-1 null T cells showed remarkable improvement of therapeutic efficacy against mouse melanoma and leukemia (Wei L. et al., 2019). Newly potential immunotherapeutic targets which have been identified by CRISPR/Cas9 screening technology are summarized in Table 3.

## Perspectives and Challenges

### Perspectives: CRISPR/Cas9 and TILs Based Adoptive Cell Therapy

The presence of tumor-infiltrating lymphocytes (TILs) in the tumor microenvironment is regarded as an adaptive anti-tumor immune response. About 67% TILs grown from metastatic melanoma were identified to be tumor specific (Goff et al., 2010). Due to the natural TCR specificity to tumor antigens, TILs have been exploited as an adoptive cell therapy (ACT) to treat cancers. Tumors were resected and fragmented into small pieces at about 1-2 mm^3^, or enzymatically digested as single cell suspension. TILs were isolated and expanded *in vitro* at the presence of interleukin-2 (IL-2) to large numbers, and then adoptively transfer to patients (Dudley et al., 2003). The clinical responses with TIL therapy were firstly demonstrated in melanoma with objective response rate (ORR) up to 72%, in which 40% patients experienced durable clinical responses and 10-20% patients reached complete remission (CR) (Rosenberg et al., 1994, 2011). The encouraging clinical responses of TILs based ACT in melanoma have stimulated researchers to conduct studies in other types of solid cancers. Three out of nine patients with metastatic cervical cancer experienced objective tumor responses (with two complete remission and one partial remission) after receiving a single infusion of autologous TILs (Stevanovic et al., 2015). Besides, ACT with TILs targeting tumor neoantigens encoded by mutated genes has achieved substantial objective clinical responses in patients with metastatic cholangiocarcinoma (Tran et al., 2014), breast cancer (Zacharakis et al., 2018), and colorectal cancer (Tran et al., 2016).

Despite promising clinical outcome have been achieved, the majority of patients with epithelial cancers did not respond to TIL therapy. The impairment and exhaustion of TILs may account for the poor responses, as TILs may be in “progenitor exhausted” state before being expanded *ex vivo*. T cells get into terminal differentiated state after rapid expansion. Studies have found that longer duration of responses to immune checkpoint blockades could be noticed in patients with melanoma who had a higher percentage of progenitor exhausted TILs (Miller et al., 2019). Progenitor exhausted TILs can respond to checkpoint blockades, while exhausted TILs cannot. TIL phenotype analysis has also revealed that less differentiated CD39^–^CD69^–^ stem-like TIL phenotype was associated with complete tumor responses and longer TIL persistence in patients who received TIL therapy (Krishna et al., 2020). Thus reversing dysfunctional T cells state and retaining stem-like TIL phenotype by using CRISPR/Cas9 gene-editing tool may further improve the efficacy of TIL therapy. Metabolism-associated factor Regnase-I was found to be a negative regulator of T cell anti-tumor responses. Knocking out Regnase-I reprogrammed T cells to long-lived effector cells with better infiltration and persistence in tumor microenvironment, and improved ACT therapeutic efficacy (Wei L. et al., 2019). Another study identified that Gata-3, a zinc-finger transcription factor, drove CD8^+^ TILs dysfunction. Disrupting Gata-3 in naïve CD8^+^ T cells improved T cells anti-tumor function (Singer et al., 2016). However, studies on engineering TILs to enhance T cells function and proliferative activity by using CRISPR/Cas9 system were rarely reported. Only zinc finger nuclease was once reported to target the gene encoding human PD-1 in melanoma tumor-infiltrating lymphocytes. The PD-1 knockout TILs could be expanded into clinical scale. In addition, the edited TILs showed enhanced *ex vivo* effector function and a significantly increased cytokine releasing compared to unedited TILs (Beane et al., 2015).

### Challenges: CRISPR/Cas9 Technology in Clinical Translation

Although CRISPR/Cas9 system have shown immense potential for improving efficacy of immunotherapy, several concerns related to safety and efficacy impede its translation to clinical applications.

Firstly, clinical efficacy of ACT based cancer immunotherapy, including CAR-T cells, TCR-T cells and TILs therapy, is dependent on adequate T cells for refusion. However, most of CRISPR-engineered T cells for clinical trials were transduced by electroporation which might result in cell damage and impeding T cell proliferation *ex vivo* (Lu et al., 2020; Song et al., 2021). Therefore, more safe and efficient delivery ways such as viral vectors are in urgent exploration. In addition, direct delivery *in situ* or *in vivo* may be an alternative option that is worthy to develop (Song et al., 2021). However, the immunogenicity of cas9 proteins may be another challenge that constrain the clinical translation of CRISPR/Cas9 system. Anti-SaCas9 (Cas9 from S. aureus) and anti-SpCas9 (Cas9 from S. pyogenes) antibodies were detected in 78% and 58% of donors, respectively. Moreover, 78% and 67% of donors possess T cells against SaCas9 and SpCas9 protein, respectively (Charlesworth et al., 2019). This showed that there were pre-existing adaptive immune responses to Cas9 proteins in human which may cause adverse effects when treating patients with CRISPR/Cas9 system. Whereas no potential rejections were noted in clinical trial (Stadtmauer et al., 2020). Some researchers point out that the pre-existing immune responses to Cas9 proteins do not appear to be an obstacle to clinical application of CRISPR/Cas9 system (Stadtmauer et al., 2020).

Secondly, although various approaches have been reported to improve gRNA design (Bin Moon et al., 2018; Matson et al., 2019; Han et al., 2020) and increase the specificity of Cas enzyme (Kleinstiver et al., 2016; Slaymaker et al., 2016), the risk of off-target effects, resulting from non-specific cutting and further leading to unwanted mutations, still remains a major obstacle to translation of CRISPR/Cas9 system to clinical therapeutic use (Cook and Ventura, 2019). Whereas, in clinical trials, no significant or only a small number of off-target sites, chromosomal rearrangements or long-range deletions were actually detected. Moreover, the frequency of off-target sites declined over time (Roth et al., 2018; Xu et al., 2019; Lu et al., 2020). But experience with more patients and longer follow-up are needed to further validate the safety and feasibility. The commonly used off-target effects detecting methods including the T7 Endonuclease I (T7E1) mutation mismatch assay, deep sequencing, and whole genome sequencing are not perfect (Ren and Zhao, 2017; Li et al., 2019). More sensitive, accurate and practical approaches are needed to identify off-target mutations. However, the past decades have witnessed the introduction and development of high-throughput sequencing and multi-omics analysis. The combination of multi-omics analysis may help detect and understand causal mutations, functions of these genes and affected cellular and signaling pathways, so as to predict the potential and significance of off-target effects.

## Author Contributions

XO and QM wrote the first draft of the manuscript. WY completed the references collection and drew the figures. XM and ZH reviewed and revised the original manuscript. All authors commented on previous versions of the manuscript.

## Conflict of Interest

The authors declare that the research was conducted in the absence of any commercial or financial relationships that could be construed as a potential conflict of interest.
